# ISAAC - InterSpecies Analysing Application using Containers

**DOI:** 10.1186/1471-2105-15-18

**Published:** 2014-01-15

**Authors:** Herbert Baier, Jörg Schultz

**Affiliations:** 1 Department of Bioinformatics, Biocenter, University of Würzburg, Am Hubland, Würzburg 97074, Germany

**Keywords:** Teamwork, Gene sets, Explorative analyses, Cross-species analyses

## Abstract

**Background:**

Information about genes, transcripts and proteins is spread over a wide variety of databases. Different tools have been developed using these databases to identify biological signals in gene lists from large scale analysis. Mostly, they search for enrichments of specific features. But, these tools do not allow an explorative walk through different views and to change the gene lists according to newly upcoming stories.

**Results:**

To fill this niche, we have developed ISAAC, the InterSpecies Analysing Application using Containers. The central idea of this web based tool is to enable the analysis of sets of genes, transcripts and proteins under different biological viewpoints and to interactively modify these sets at any point of the analysis. Detailed history and snapshot information allows tracing each action. Furthermore, one can easily switch back to previous states and perform new analyses. Currently, sets can be viewed in the context of genomes, protein functions, protein interactions, pathways, regulation, diseases and drugs. Additionally, users can switch between species with an automatic, orthology based translation of existing gene sets. As todays research usually is performed in larger teams and consortia, ISAAC provides group based functionalities. Here, sets as well as results of analyses can be exchanged between members of groups.

**Conclusions:**

ISAAC fills the gap between primary databases and tools for the analysis of large gene lists. With its highly modular, JavaEE based design, the implementation of new modules is straight forward. Furthermore, ISAAC comes with an extensive web-based administration interface including tools for the integration of third party data. Thus, a local installation is easily feasible. In summary, ISAAC is tailor made for highly explorative interactive analyses of gene, transcript and protein sets in a collaborative environment.

## Background

Over the last 10 to 15 years, biology has changed into a ‘more precise and quantitative science’ [1]. New high throughput technologies generate data covering different aspects of molecules in an ever increasing pace. As a result, we are now drowning in data when looking for biological stories. Accordingly, bioinformatics methods and databases to deal with this flood of information have been developed. Whereas in the beginning these computational tools were available mainly to bioinformaticians, many tools and databases are nowadays accessible via the web and can be interrogated also by non-computational trained biologists. But, there are still some challenges to cope with when trying to find biological meaning within this flood of data. First, different types of data are distributed over a wide variety of databases and web-based resources. For example a biologist will have to go to Ensembl [2] or the UCSC genome browser [3] when searching for genomic information. Next she might look up functional information in the GeneOntology [4] (which generously has been integrated in a multitude of other tools and databases). If especially interested in disease genes, OMIM [5] and DrugBank [6] might be useful resources. Next, to identify functionally related genes, databases like KEGG [7], STRING [8], or in more specific cases mirRBase [9] might be questioned. The challenge of distributed data has been addressed by different higher level tools. These focus mainly on the evaluation of larger datasets generated by high throughput methods and the more or less automated annotation and statistical evaluation of these gene sets. An outstanding example is DAVID [10,11]. It provides a variety of functional annotation tools (like gene enrichment analysis, pathway mapping), gene accession conversion, a genome browser and a stateful web service [12]. Gene lists can be uploaded in different identifier formats and sub lists can be created during the enrichment analysis. Furthermore, the lists can be renamed, removed, combined and downloaded. Related functional profiling tools are GEPAT [13], Onto-Express [14], Onto-Tools [15], FACT [16] BABELOMICS [17], FatiGO + [18], GeneTrail [19], g:Profiler [20], VisANT [21], Reactome [22], MAPPFinder [23], GFINDer [24], GOLEM [25]. Provides a good overview on enrichment tools [26]. The Ingenuity System [27] is a commercial software that is widely used to analyze and model complex biological and chemical systems. Finally, Cytoscape [28] is a generic tool for network analysis and visualization whose network information can be associated with gene expression data.

As mentioned above, the main goal of these tools is the statistical evaluation and functional characterization of given, mostly large, gene sets. Thus it is in the nature of these tools, that the user is not allowed to interactively change the gene lists within one analysis. For their application, this makes perfect sense, as these tools provide a reproducible annotation pipeline. But, there is a different type of user who might be more interested in the explorative analysis of smaller gene sets. She might start with a few genes, analyze them under one aspect and find other genes of interest. Now she might want to extend the gene sets and analyze the new list under a different aspect. WebGestalt [29,30] did a first step into this direction. Here, different sets could be merged, but the manual addition of genes is not possible. However, in the current online version of WebGestalt these set operations are missing. Complementary, WhichGenes [31] enables generating gene sets based on various sources and to combine these sets. Thus, sets of genes involved in glycolysis and encoded on a specific chromosome can be generated. Still, it does not allow viewing one gene or gene set under different biological aspects or performing analyses on sets. Thus, we wanted to create a tool which integrates the main idea of enrichment tools, namely to analyze gene lists under a wide variety of functional aspects, with the ability to manually add and delete sets of interesting genes to enable explorative analyses. As the amount and detail of functional information differs widely between different species, we also wanted to enable cross species analysis. We have implemented these ideas in the Web based tool ISAAC (http://isaac.bioapps.biozentrum.uni-wuerzburg.de), an acronym for ‘InterSpecies Analysing Application using Containers’.

## Implementation

### Object oriented strategy

**Figure 1:**
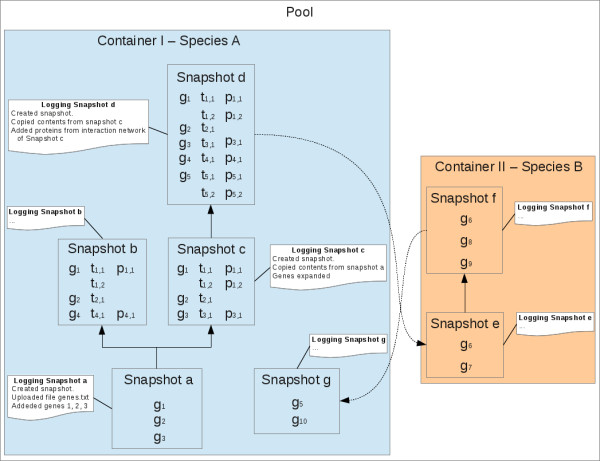
**ISAAC core concepts – Let*t***_***a,b***_**be the transcript coding for the protein*p***_***a,b***_**and*g***_***a***_**be the gene coding for the transcript*t***_***a,b***_**.** A user creates the snapshot a in the container I for species A and uploads a file containing three genes, namely g_1_, g_2_ and g_3_. From snapshot a, the user creates two child snapshots b and c (the contents of the snapshot a are replicated to the snapshots b and c). In the snapshot b the user adds the proteins p_1,1_ and p_4,1_ (due to the transitive rule, t_1,1_, t_4,1_ and g_4_ are also added) and the transcripts t_1,2_ and t_2,1_ and removes the gene g_3_. In the snapshot c the genes are extended, this means their transcripts and proteins are added. From snapshot c the child snapshot d is created. All proteins of the interaction network containing all proteins which directly interact with all proteins in the snapshot c, namely p_1,1_, p_1,2_, p_3,1_, p_4,1_, p_5,1_ and p_5,2_ are added. Again, corresponding transcripts and genes are added automatically. Next, the snapshot e in the container II for species B is created and the orthologous genes of snapshot d are imported. From snapshot e the user creates the child snapshot f and adds the genes g_8_ and g_9_ and removes the gene g_7_. Finally, the snapshot g is created in the container I and the orthologous genes of snapshot f are included.

Traditionally, the selection of gene sets follows a procedural approach. An example might be the BioMart interface to the Ensembl databases [32]. In the most straightforward scenario, the user first selects a species/dataset, then defines filters for the gene sets and chooses which attributes should be reported. Finally, the corresponding set is calculated. If the user wants to add other filters, the procedure has to be changed and a new set is calculated. Contrasting ISAAC uses a more object oriented strategy. Its central point of view is the information that the biologist wants to analyze and his/her knowledge. The information is represented as sets which can contain genes, transcripts and proteins (the objects) which then can be compared and/or modified (the methods) (Figure 1). All elements in a set belong to the same species and the following transitive property is satisfied: if a protein belongs to a set, then the coding transcript belongs to the set, and if a transcript belongs to a set, then the coding gene belongs to the set. Formally, let *p* be a protein, *t* be a transcript and *S* be a set of genes, transcripts and proteins of a species, then

$$\begin{array}{c} p\in S\to t_{p}\in S\\ t\in S\to g_{t}\in S \end{array}$$

where *t*_*p*_ is the transcript of the protein *p* and *g*_*t*_ is the gene of the transcript *t*. The reverse property is not required, i.e. a gene can be part of the set without adding its transcripts or proteins. It is assured that the sets are consistent at all times. Biologically, this transitivity is of importance as it enables the integration of information focusing on different biological entities. A user can add a specific splice variant of a gene, i.e. a protein, to a set. Automatically, the corresponding transcript as well as the coding gene is added to the set. Therefore, information related to the gene like a disease association can be analyzed. Still, when going back to the protein level only the specific isoform is considered. Complementary, if the user adds a gene to a set no transcript or protein information is added by default as further information might be specific to one or a few of the isoforms of the gene. If desired, all transcripts and proteins encoded by a gene can be added to the set, thus ensuring the consistency of the sets. The basic set comparisons (equal =, proper subset ⊂, subset ⊆) and operations (union ∪, intersection ∩, set minus \) are supported. Hence, elements can be added using the union operator and removed using the set minus operator. Due to the transitivity, the set comparisons and the set minus operation are performed on a selected level, namely genes, transcripts or proteins. Additionally, sets can be created, copied, cleared, removed, imported and exported.

A version control system manages the sets in a tree structure that allows biologists to keep track of different versions. Furthermore, for each action a history is logged enabling the tracing of changes of sets. In ISAAC context a set configuration managed by the version control system is called snapshot and a container is a collection of snapshots. Each snapshot belongs to exactly one container and all snapshots in a version tree belong to the same container. At any time, a user can go back to an older snapshot and use it as the starting point for a new analysis by generating a new child snapshot. Thus, a tree like structure of analyses can be generated. Special properties can be defined in a container, such as a description, color and comments.

This core system can now be used from different modules, which mainly perform analyses on sets, modify sets with the given methods and visualize sets and results of analyses. Thus, complex biological analyses covering different biological aspects are broken down into independent, interchangeable modules. The resulting non-linear application flow supports the biologist in searching for biological stories in their data.

### Java EE technology

ISAAC is implemented in Java EE 6 (Java Platform, Enterprise Edition) and uses the web component JSF 2.0 (Java Server Faces) to generate dynamic web pages with Ajax support. This technology substantially simplifies the development of an application, since it creates standardized, reusable modular components and enables the tier to handle many aspects of programming automatically like persistence, messaging and security. Hence, a Java application server is required to run ISAAC. ISAAC was developed and tested using JBoss application server. The strict client/server architecture allows multiple frontend clients to be developed and integrated in a standard and easy way, since the process logics are performed on the server. In ISAAC, there is no time out for a client web session. As long the web page is open, its session is held on the web server.

As aforementioned each module contains everything necessary to perform the desired functionality and, therefore, information has to be imported from other sources. However, this information is not tied to specific sources. Each module provides well-defined interfaces and any source fulfilling their requirements can be used. The development of new modules and even web services is therefore straightforward.

Module processes requiring large computer resources are started in background, which avoids blocking the clients till the processes are finished. As soon as a process is finished, the owner is notified within the web interface or, if desired, also via E-Mail. The processes’ results are held in the private pools and can be recovered as needed.

### Team work capabilities

Today, many research groups are embedded in larger teams. Frequently, different groups work on related aspects of a biological phenomenon using different model species. Therefore, ISSAC as a multi-user system supports teamwork. Each user owns private pools of (i) containers with sets of genes, transcripts and proteins and (ii) results of analysis. Furthermore, pools shared within a group of users can be created. Access to the pools comes in two authorization levels: (i) in the normal level a user is only allowed to read the pool data and (i) in the coordinator level a user is also allowed to update pools and to add/remove users from the group. Special groups can be defined, which allow all users to access their pools in a normal level. In addition to sharing containers, coordinators can also place processes in group pools enabling access to all group members.

## Results and discussion

### Available modules

An overview of currently implemented modules is given in Table 1. Obviously, the core of a system to analyze lists of genes has to hold information about genes, transcripts and proteins. This is handled in the **genome module**. On start the chromosomes of the selected species are displayed. Here, regions can be selected and genes within this region are shown. These can either directly be added to a snapshot or further inspected in the ‘gene view’. Here, genes, transcripts, proteins, associated diseases, drugs and miRNAs are shown, among other information. Transcripts and proteins can be selected and added to snapshots. For proteins, Interpro annotations are provided, allowing a first functional characterization [33]. Snapshots can be selected and their contents will be highlighted in the chromosome view using the respective container color. As ISAAC also provides information about orthologous relations, the user can switch between different species automatically ‘translating’ the gene sets. Further genomes and enzyme activities can be imported from Ensembl [2,34] and UniProt [35], respectively, via the administration interface.

**Table 1:** Currently implemented modules

**Module**	**Datasource(s)**	**Usage**
Genome	Ensembl [2], UniProt [35]	Search for genes, transcripts and proteins and add them to sets. Features are visualized.
Protein Interaction	STRING [8]	Analyze protein interactions within a set. Identify interacting proteins and add them to sets.
Pathway	KEGG [7]	Analyze genes in sets in a metabolic and pathways context. Add further genes of a pathway to sets.
GO enrichment	GeneOntology [4]	Functional characterization of sets. Extend sets based on function.
microRNA	TarBase [38]	Reveal microRNA based regulation of genes in sets. Search for genes regulated by specific microRNAs.
Disease	OMIM [5]	Identify mendelian disease genes in sets. Search for genes associated with a mendelian disease.
Drug	DrugBank [6]	Identify drug targets in sets. Search for genes affected by a drug.
Orthology	Ensembl [2]	Orthology based translation of sets to other species.
Team Work	-	Share sets between users and within groups.

In a cell, no protein works on its own. Thus, to understand the function of a single or sets of proteins, one always has to consider their interaction partners. To allow analyzing sets in this context, we implemented the **protein interaction module**. Starting with direct interactions, it can be extended to show higher level interactions. Although data from any protein interaction database can be integrated, we currently imported data from STRING [8]. Thus, interactions are annotated on the gene level, although in the cell proteins are interacting. As ISAAC ensures consistent sets with gene and transcript automatically added, this distinction is hidden from the user. In the graph, single genes of interest or all genes can be added to a snapshot. Again, STRING data can be imported via the administration interface.

The protein interaction module enables viewing sets in the context of networks. Still, the type of interaction is not detailed out. The biological **pathway module** allows the graphical visualization of enzymes belonging to a snapshots of a species in their pathway context [7]. In a pathway diagram the EC-numbers are highlighted in three different ways: (i) the enzymatic function is covered by the selected snapshots, (ii) the species contains an enzyme with the EC classification but it is not part of the snapshots and (iii) no protein with the EC number is annotated in the species. As usual, genes coding for a given enzymatic function or the whole pathway can be added to snapshots. Furthermore, an enzyme can be selected going to the protein’s view of the genome module.

To enable a fast functional characterization of genes, transcripts and proteins in sets, we implemented the **GO term enrichment module**. Based on GeneOntology [4], the biological processes, cellular components and molecular functions of proteins/genes of snapshots can be analyzed and displayed. To improve the tree based presentation of the directed acyclic GO graph, nodes with more than one parent are replicated and only sub trees with at least one match are shown. For each node the following information is given: (i) the GO description, (ii) the number of proteins in the selected snapshot(s) belonging to this node, (iii) the total number of proteins in the genome belonging to this node, (iv) the number of proteins of the selected snapshot(s) belonging to the sub tree rooted in this node, (v) the total number of proteins in the selected snapshot and (vi) the p-value of this node (parent–child-union approach of the hypergeometric distribution [36,37]). Proteins belonging to a node or its sub tree can be added to snapshots. On the other direction, for each selected protein a list of GO identifiers is given. A protein can be selected going to the protein’s view of the genome module. Furthermore, a GO identifier can be selected which highlights the paths to the root (a node can coexist more than once in the tree). All GO data can be imported via the web based administration interface.

To get insights about possible regulatory mechanisms of genes in a snapshot, the **microRNA module** was implemented. It supports the search for microRNAs and lists genes regulated by the specified microRNA, which can be added to a snapshot. Complementary, all microRNAs regulating genes in selected snapshots can be listed. Information about microRNA was imported from TarBase [38].

Finally, we enable to search for genes associated with a disease and genes which are known drug targets in the **disease module** and the **drug module**, respectively. Again, a user can start with a disease or a drug, get information about involved genes and add them to the snapshot. Alternatively she can list all diseases associated with genes in snapshots and drugs affecting these genes. The disease module supports external links to the OMIM database [5] and the drug module to DrugBank [6]. Tools are provided to import OMIM information from the Ensembl (BioMart) database and drugs from DrugBank.

One of the main ideas behind the development of ISAAC was to carry out analyses across species boundaries. This is enabled by adding **orthology** information. Here, the user can switch between different species and the actual snapshot is ‘translated’ to the new species. For administration, an interface was implemented to import orthologous data from TSV files created by e.g. BioMart [34].

### Use cases

#### Different aspects of genes/proteins

**Figure 2:**
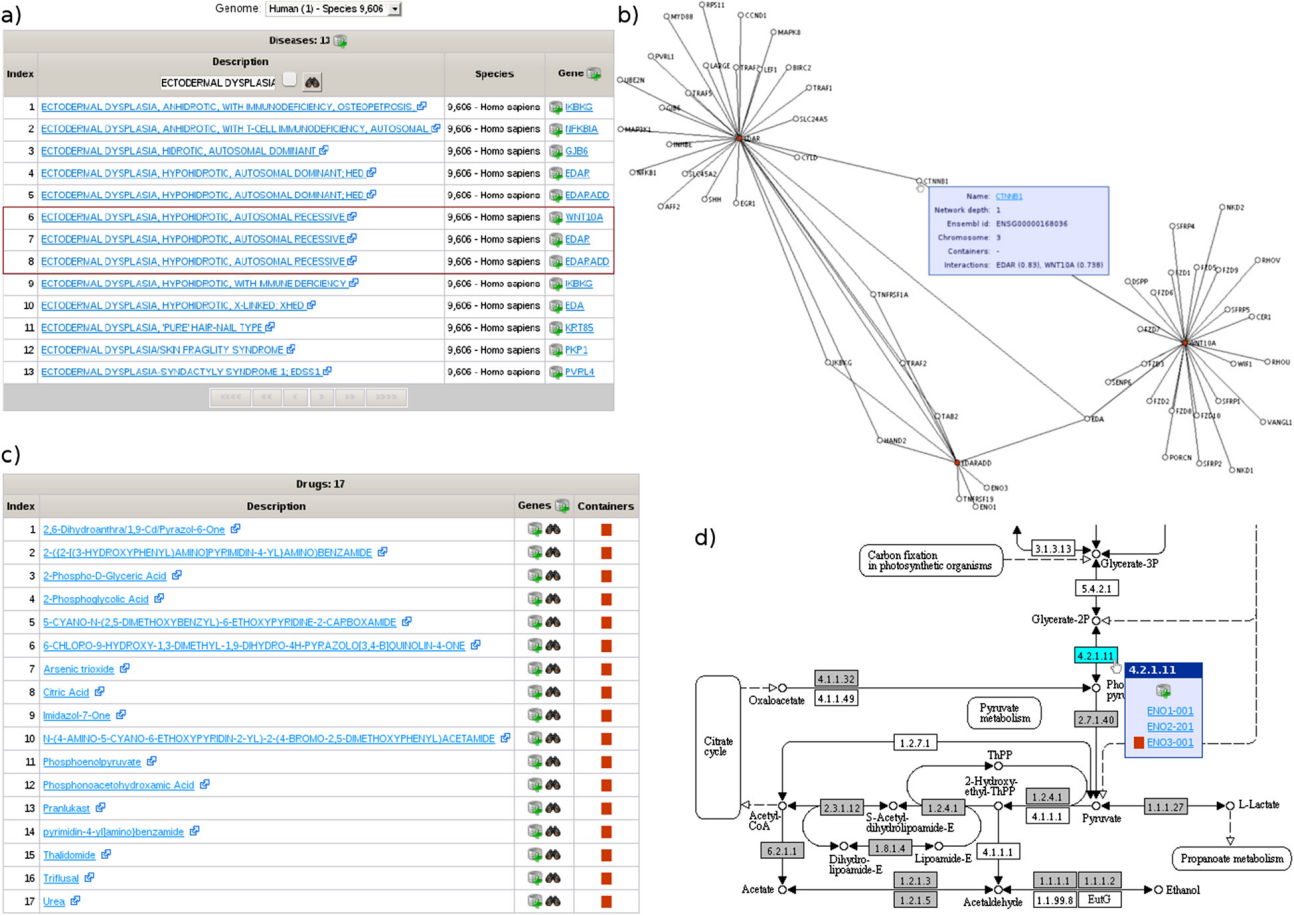
**Analyzing different molecular aspects of genes and proteins. a)** Identification of disease genes – The user is interested in the disease Ectodermal dysplasia. From the returned list she focuses on autosomal recessive hypohidrotic ectodermal with three associated genes (red enclosed region). **b)** Interacting proteins – The graph shows the interaction network containing all genes which directly interact with the three genes in the snapshot. This interaction network comprises 58 genes. **c)** Identification of drug targets – 7 of the interacting genes are targets of known drugs. **d)** Integration into metabolic networks – One of the identified genes is involved in Glycolysis / Gluconeogenesis. Enzymes present in the snapshot are highlighted turquoise. Other enzymes present in the human genome are highlighted grey. Enzymes absent from the genome are not highlighted.

As ISAAC includes information from OMIM, a user can look up a disease, for example ectodermal dysplasia. OMIM Entries and the associated genes are displayed (Figure 2a). Focusing on the recessive autosomal variant, she creates a snapshot with the three associated genes. In a first attempt to search for drug candidates, she can switch to the drug tab and list all known drugs which interact with proteins encoded by genes in the actual snapshot. Here, she will find none. In an attempt to search for further candidate genes, she switches to the protein interaction module. Here, she creates an interaction network containing all genes which directly interact with all genes in the current snapshot, i.e. the disease genes (Figure 2b). In the example case, this network comprises 58 genes, which are added to the snapshot. Now, with this expanded gene set, one can go back to the drug tab and check for drug-able genes. Indeed, one finds that 7 of the directly interacting genes are targets of known drugs (Figure 2c). These now identified candidate genes can be added to a new snapshot. To predict side effects, one can go to the pathway tab and check, in which pathways the genes are involved. Here, one finds one gene, which is involved in glycolysis (Figure 2d). After creating a new snapshot with this gene, one can switch species and identify the mouse ortholog as a candidate mouse model. Obviously, this scenario is rather naïve considering the identification of drug candidate genes, but it should exemplify the possibilities to view gene lists under different biological aspects and how a user can interactively adapt the gene/protein sets.

#### Non-Model organisms

**Figure 3:**
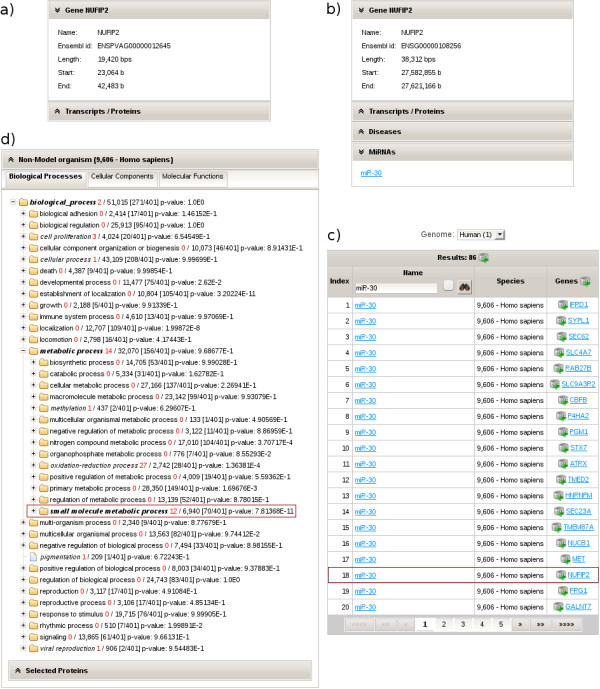
**Working with non-model organisms. a)** The user is interested in the bat gene *NUFIP2*. **b)** The panel displays the human ortholog and shows, that it is regulated by the microRNA miR-30. **c)** The table lists the 86 human genes regulated by the miRNA miR-30. **d)** 20 of the 86 genes regulated by miR-30 are overrepresented in the GO biological process classification “small molecule metabolic process” with a p-value: p = 7.81368x10^-11^. 19 bat genes are orthologous to the 20 human genes regulated by the microRNA miR-30 and involved in the specific function “small molecule metabolic process”, namely *ADPGK, ATP2A2, DOCK7, ELMOD2, GNAI2, GPD2, IDH1, JUN, MAT2A, NPR3, NT5C3, NT5E, P4HA2, PNP, PPP2R4, RAB27B, TMED2, UAP1* and *WNT5A*.

The increasing pace of genome sequencing results in genomes of experimentally poorly characterized species. As an example, we integrated the genome of the large flying fox (*Pteropus vampyrus*) into ISAAC. Again, a researcher might start with a single bat gene of interest, e.g. *NUFIP2* (Figure 3a). To get a first glimpse of the function of this gene she can switch to a better understood organism like human (Figure 3b). Here, she can go to the microRNA module to check, whether this human ortholog of the bat gene is regulated by a microRNA. Indeed, she will find that the human *NUFIP2* gene is regulated by miR-30. The snapshot can now be enlarged by all other genes which are regulated by this microRNA, here 86 genes (Figure 3c). To see which functions are regulated by this miRNA, one can go to the GO enrichment module and search for overrepresented GO classifications (Figure 3d). In the example case, the user might focus on ‘small molecule metabolic process’ (p = 7.8×10^-11^). The researcher might switch the focus from miRNA to this defined function and add the genes to a new snapshot. An intersection with the previous snapshots enables to home in on genes which are regulated by miR-30 and involved in small molecule metabolism (20 genes). Finally, she translates this intersection back into the bat, resulting in 19 candidate genes which might be regulated by a microRNA and involved in small molecule metabolism. From here on, she could design an experiment to test, whether an ortholog of the human microRNA is indeed found in the bat and, if this is the case, whether the orthologous genes are indeed regulated by this microRNA.

#### Team work

**Figure 4:**
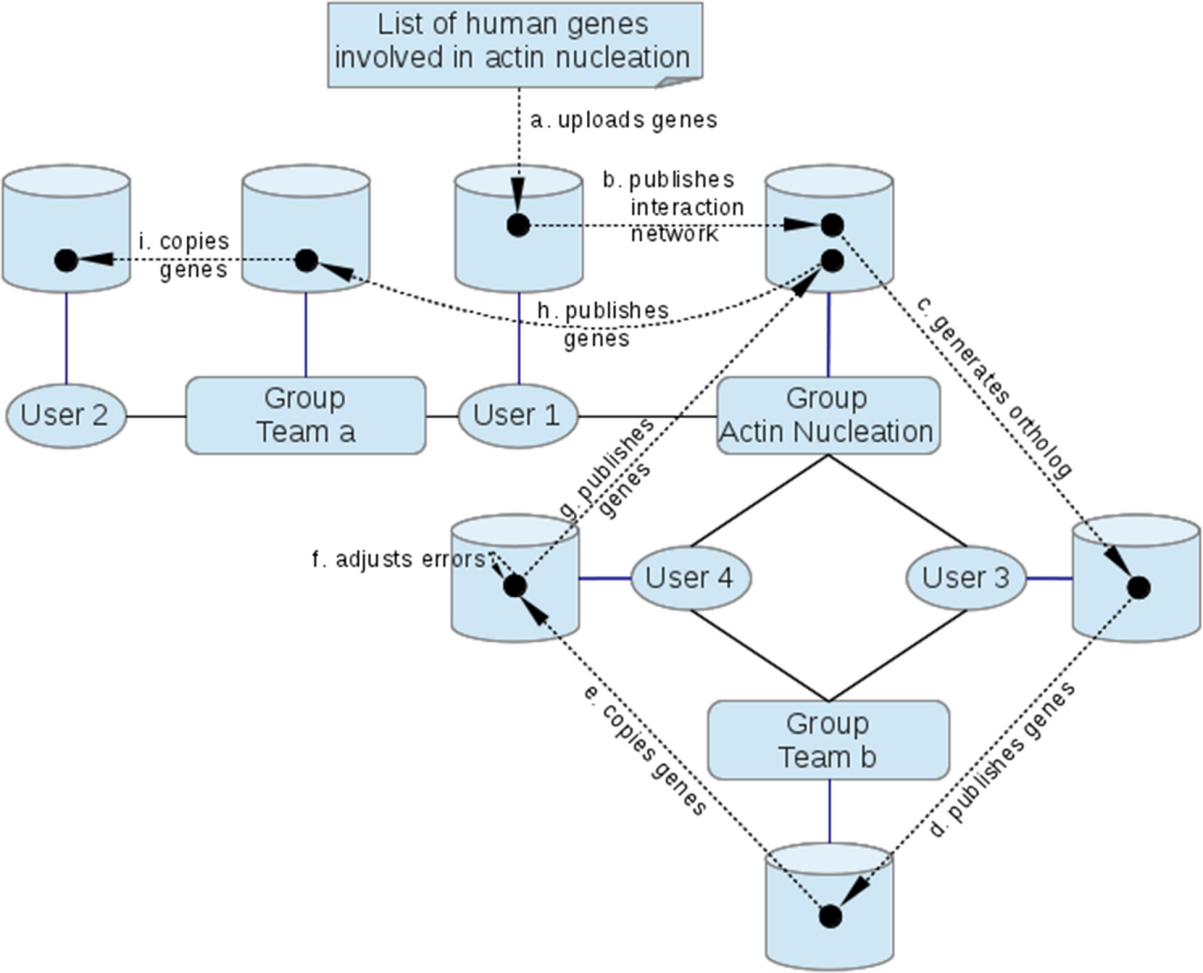
**Working in teams.** The diagram shows users and groups with their respective pools (blue lines). The users 1 and 2 are members of “Team a” with user 1 as coordinator. The users 3 and 4 belong to the group “Team b” and here user 3 is the coordinator. Furthermore, users 1, 3 and 4 are part of the group “Actin Nucleation” (users 1 and 4 are coordinators). The continuous black lines denote the memberships. Following the dashed arrows, user 1 imports into its private pool a manually curated list of human genes involved in actin nucleation **(a)** and publishes its protein interaction network to the group “Actin Nucleation” **(b)**. Using orthology mapping user 3 translates the interaction network into another species **(c)** and publishes it to the group “Team b” **(d)**. User 4 copies this list to his private pool **(e)**, manually curates it **(f)** and publishes this new list to the group “Actin Nucleation” **(g)**. The new list is published to group “Team a” by user 1 **(h)** and user 2 copies it to his private pool **(i)**.

Today, biological research is only rarely performed by a single lab on its own. In most cases, a lab works closely together with others to study different aspects of a gene using a wide range of techniques and organisms. The team working features of ISAAC might simplify the communication across different labs. As an example assume a consortium of groups working on actin nucleation (Figure 4). As a starting point, one researcher has deposited a manually curated list of human genes involved in actin nucleation. Within ISAAC a user group with all researchers of the consortium was created. Now the list can be published such that the whole group (and only this group) has access to the list. In addition to gene lists, also processes, i.e. results calculated within modules, can be published. For example, a user can publish an interaction network of the actin nucleation genes and all direct neighbors. A researcher of another group, working with a different species can now import the interaction network into her private pool. From here on she can use all features of ISAAC like mapping the genes into a new model species. Again, this result can be published, imported by another user who might manually curate this set and publish the results. Thus, the knowledge about gene sets of interest can be easily distributed within the consortium.

## Conclusion

ISAAC enables non-computer trained researchers to explore gene lists under different biological aspects. From a user’s point of view, the main difference to other related projects is that the gene lists can be changed interactively at any point of an analysis. Obviously this inherently carries the danger of losing track about how a set was generated. We therefore implemented an integrated version and logging system which supports the users on the persistence, administration and tracking of these sets. Together with the snapshot function, every part of the analysis can be traced and become a starting point for new analyses. Thus, ISAAC indeed enables the explorative mining for genes of interest. As new genomes are sequenced with an increasing pace, analyses crossing the species border become of increasing importance. As ISAAC includes information about orthologous relationships between genes, users can switch between species, automatically ‘translating’ gene sets from one species to another. Finally, ISAAC is not focused on single users. Instead, it offers options to share sets and even results of analyses between users and teams. Thus, not only a single user can look at a problem from different biological views, she can also let other researchers look at her genes to get an external view. Thereby, ISAAC supports multi team collaborative efforts getting ever more prominent in biological research.

From a programmer’s point of view, ISAAC is based on an object oriented approach contrasting more workflow oriented programs, which are usually procedural. Sets of proteins, transcripts and genes with a well-defined structure together with comparison and operation methods build the core of this tool. Using this core, different modules can be implemented covering different biological aspects. The object oriented strategy and its modularity make this straightforward. Especially when performing highly explorative analyses, a user will need some breaks to e.g. gather further information. Therefore, there is no time out for a client web session. As long the web page is open, its session is held on the web server. To enable the integration of further modules, the source code is freely available from our web page.

Together with the web client, we developed an administration interface. Here, not only users and groups can be managed. More importantly, integration of third party data needed by a module can be carried out via the administration interface. This allows for example the straightforward addition of further genomes, as scripts which directly can insert Ensembl genomes are implemented and can be administrated via the web interface.

In summary, with its focus on small but highly explorative analyses ISAAC closes the gap between databases covering only on one or a few aspects of genes and proteins on the one hand and automated analysis tools which do not allow for interactive modifications of gene lists on the other.

## Availability and requirements

**Project name:** ISAAC.

**Project home page: **http://isaac.bioapps.biozentrum.uni-wuerzburg.de.

**Operating systems:** Platform independent, tested on linux.

**Web browser:** Tested with Mozilla Firefox 16.0.2 and Internet Explorer 10.

**Programming language:** Java ≥ 1.7.

**Other requirements:** Java application server (Java EE 6 and JSF 2.0).

**License:** Free for academic users under the GNU Lesser General Public License (LGPL).

## Competing interests

The authors declare that they have no competing interests.

## Authors’ contributions

HB developed and implemented the system. JS designed and supervised the project. Both authors wrote, read and approved the final version of the manuscript.
